# Georgia Medical Asssociation

**Published:** 1874-05

**Authors:** 


We gladly welcome to the Gate City of the South Judge D. M. Hood, of Rome, an old veteran of the periodical press, who has always displayed the abilities of a trusty commander in keeping his ship fairly abreast the waves as long as human skill can avail, and, being the first to discover an irreparable breach, in safely landing his passengers and crew before a foundering barque goes down. His accession to the business management of the *Commonwealth* "means business," and with the well-established reputation of Col. B. F. Sawyer—as chivalrous, bold and conscientious in his thrusts now with the pen as he was in years gone by with the sword—we look for a career of high prosperity for the *Commonwealth.*

